# Perceptions of Conflict at the Transition to Parenthood: Exploring Adult Attachment Pairings as Predictors of Emotional Flooding

**DOI:** 10.1111/famp.70057

**Published:** 2025-07-30

**Authors:** Sean D. Morgan, Erica M. Woodin

**Affiliations:** ^1^ Department of Psychology University of Victoria Victoria British Columbia Canada

**Keywords:** adult attachment, conflict, couples, emotional flooding, transition to parenthood

## Abstract

Understanding how attachment styles between partners relate to the dysregulation of emotions during couple conflict has received little attention, especially over the transition to parenthood. This research investigated how combinations of expectant couples' attachment styles jointly predict *emotional flooding*, which is a form of interpersonal emotion dysregulation. Using a sample of 98 mixed‐gender couples residing in Canada, we used multilevel modeling to examine actor effects (e.g., one's attachment insecurity predicting their own flooding), partner effects (one's attachment insecurity predicting flooding in their partner), and interactions between partners to examine its association with emotional flooding at the third trimester of pregnancy and across early parenthood. Longitudinally, couples were followed from the third trimester to 4 years postpartum to explore how attachment pairings predicted changes in flooding across parenthood. Attachment anxiety in men predicted their own propensity to become flooded during conflict, as well as their partner's flooding. An interaction was seen at the third trimester, such that men who were avoidantly attached reported greater flooding when their partner was high in anxiety compared to low in anxiety. Finally, men's flooding was associated with greater increases over time when high avoidance in men was paired with low avoidance in women, whereas flooding showed the smallest increase when both partners reported low avoidance. Findings suggest that the fit between each partner's attachment styles can improve understanding of the emotional mechanisms experienced during conflict, especially during the often‐stressful period of early parenthood.

## 1 Introduction

The transition to parenthood is a period of readjustment for the family system and constitutes a period of stress and change at both the individual and dyadic level (Miscioscia et al. 2021). The experiences of parenthood vary between couples and individuals; while some couples note positive gains over this period, other couples report negative changes, such as increased conflict and decreased intimacy and other positive displays (Kluwer 2010). While research examining couple conflict has identified numerous risk factors such as attachment insecurity (Curran et al. 2005) and forms of emotion dysregulation (Ben‐Naim et al. 2013), understanding these factors in tandem across the transition to parenthood is limited (Rutherford et al. 2015). Moreover, the literature is fraught with different measures of emotion dysregulation that solely examine the intrapersonal facets of difficulties regulating emotions (e.g., Cheche Hoover and Jackson 2021). In contrast, *emotional flooding*, defined as a response to a partner's negative affect during conflict that is cognitively disorganizing, unexpected, and overwhelming (J. M. Gottman 1993, 1994), can be conceptualized as an interpersonal emotion regulation response. While emotional flooding has been associated with a host of negative outcomes (e.g., intimate partner violence, substance misuse; Biesen et al. 2022; Foran et al. 2020), research identifying the antecedents of flooding is virtually nonexistent. Therefore, the purpose of the current study is to understand the links between attachment orientations, which have implications for the way in which individuals manage emotions, and emotional flooding in intact couples. Our study will utilize longitudinal methods to understand whether emotional flooding is a trait or state‐like characteristic, as well as how attachment pairings between expectant couples predict the experience of flooding over the transition to parenthood.

### 1.1 Emotion Regulation From an Attachment Lens

Attachment theory posits that humans are born with an innate psychobiological system (the attachment behavioral system) that motivates proximity seeking to significant others (attachment figures; Bowlby 1973). Due to the unreliability of attachment figures, secondary attachment strategies develop, which can involve hyperactivation (e.g., proximity seeking) or deactivation (e.g., downregulation of affective states) of the attachment system (Cassidy and Kobak 1988). Psychometric research has shown that attachment styles can be measured in two independent dimensions: attachment anxiety and attachment avoidance. Individuals who are low on both dimensions are classified as securely attached (Brennan et al. 1998). Attachment anxiety is characterized by dependence and a need for closeness and reassurance from one's partner. Attachment avoidance is characterized by excessive independence and a desire to maintain emotional distance from one's partner (Mikulincer et al. 2003). Based on these strategies for seeking security through the attachment system, different ways of interacting with romantic partners emerge during times of distress for secure, anxious, and avoidant individuals (Mikulincer and Shaver 2005).

The areas of the brain that are activated during attachment‐related cognitions (e.g., hypervigilance during conflict) appear to be the same areas activated during emotion regulation processes (Coan et al. 2006). In terms of emotion regulation strategies, it has been found that anxiously attached individuals tend to perceive emotions as aligned with attachment goals, which can even lead to the exaggeration of emotions (known as distress‐intensifying appraisals; Mikulincer and Shaver 2007). Avoidant people, on the other hand, inhibit emotional states that do not align with the goal of keeping their attachment system deactivated (Mikulincer and Shaver 2007). Moreover, these strategies are used by avoidant individuals to inhibit negative emotions, such as fear, sadness, and distress, because these emotions are associated with threats and vulnerability. Overall, emotions, along with regulatory mechanisms, can be thought of as the conduit through which attachment representations and appraisals of conflict are related.

### 1.2 Attachment, Emotions, and Conflict

Rather than viewing each partner's attachment singularly, Feeney and Karantzas (2017) highlighted that conflict involves the complex dynamics of both partners' attachment anxiety and avoidance. In addition to the strategies employed by both individuals, certain attachment pairings might lead to dysregulated emotions and possibly different propensities for partners to become emotionally flooded. Currently, there are two opposing explanations for how such attachment pairings might influence dyadic emotion regulation processes.

#### 1.2.1 Interpersonal Attachment Theory

The interpersonal examination of attachment is grounded in two competing theories: the complementary hypothesis and the similarity hypothesis (Holmes and Johnson 2009). The complementary hypothesis suggests that partner preference is based on how well partners confirm attachment expectations. For example, this theory suggests that anxious individuals should prefer avoidant partners because this pairing confirms their negative expectations of partners being distant in relationships (Holmes and Johnson 2009). Avoidant individuals, on the other hand, should prefer anxious partners, confirming their negative expectations of partners being clingy and overly reliant (Holmes and Johnson 2009).

Anxious‐avoidant pairs might be particularly volatile because each style has conflicting regulation strategies. Avoidant individuals strive to maintain relational distance and independence and utilize deactivating strategies in the face of threats, such as downplaying distress (Beck et al. 2013). Anxious individuals strive to attain relational closeness and use hyperactivating strategies in the face of threats, leading to an overreliance on their partner for comfort and support (Beck et al. 2013). Dyadic analyses support this interaction, finding that emotion dysregulation increases in couples where one partner is high in attachment anxiety and the other is high in avoidance (Taylor et al. 2018). In the context of heterosexual relationships, Beck et al. (2013) found that wives' anxiety and husbands' avoidance interacted to predict husbands' distress when anticipating couple conflict.

Recent research has conceptualized a new theoretical framework to explain attachment styles in romantic partnerships. Grounded in the similarity hypothesis, which predicts that individuals will show preference toward partners with a similar attachment style to their own, the dyadic regulation process posits that the similarity in attachment styles seen between partners is implicated in relationship processes (Holmes and Johnson 2009). Support for this theory is largely based on partner preferences rather than actual couple matching (e.g., attraction to hypothetical partners; Klohnen and Luo 2003). Moreover, recent literature suggests that the impact of attachment matching on relationship functioning (e.g., relationship satisfaction) may depend on the type of insecurity. In support of the similarity hypothesis, Wang et al. (2022) found that dyads scoring equally high or low in anxiety possessed a better‐functioning relationship than dyads where only one individual was high in anxiety. This aligns with prior findings that anxiety matching occurs in married couples (Feeney 1994) and is linked to relationship stability (Conradi et al. 2021).

However, fewer studies have examined the matching of attachment avoidance, and existing support is often based on conflict styles rather than direct measures of attachment insecurity (J. M. Gottman 1999). Among studies explicitly assessing avoidance matching, findings suggest that high levels of similarity may be associated with lower relationship satisfaction (Wang et al. 2022). To date, few studies have examined how attachment pairings relate to factors beyond relationship satisfaction, such as conflict perceptions and emotion dysregulation. Understanding how attachment pairings contribute to emotional flooding may clarify mechanisms of interpersonal functioning. For example, mismatches in conflict needs, such as anxious individuals seeking reassurance while avoidant individuals prefer distance, could hinder resolution strategies and escalate emotional distress.

### 1.3 Emotional Flooding

Given the interpersonal nature of attachment pairings, the strategies through which partners regulate their emotions are also highly interpersonal. One common paradigm to assess interpersonal emotion dysregulation is through couple conflict (Malik et al. 2020). From a theoretical standpoint, strategies used by insecurely attached individuals during conflict could be viewed as dysregulated since hyperactivating (e.g., clinging, controlling, or coercive actions seen in attachment anxiety) and deactivating (e.g., avoidance, suppression as seen in attachment avoidance; Shaver and Mikulincer 2002) behaviors have each been associated with negative outcomes (e.g., diminished conflict resolution, decreased intimacy; Paquette et al. 2020). However, assessing how one regulates, or fails to regulate, their emotions in response to a partner's behaviors is an emerging area of research.

One such construct that focuses on the interpersonal dynamic of emotions during conflict is emotional flooding. Flooding in interpersonal relationships was conceptualized by J. M. Gottman (1993), who described it as a response to an intimate partner's negative emotions, feeling as though they are unexpected, overwhelming, and cognitively disorganizing. Flooding requires the encoding of a partner's behavior as aversive, and the subjective appraisal that one is unable to manage their emotions considering their partner's actions. Therefore, flooding itself is not a particular emotional experience (e.g., sadness) but is the extent to which an individual appraises their partner's emotions as overpowering (Malik et al. 2020). This conceptualization situates flooding at the intersection of emotion and cognition, given that one is affectively appraising a situation and its interfering nature (Del Vecchio et al. 2016).

Overall, flooding is thought to be an overlearned response to conflict (J. M. Gottman 1993), which might mirror the overlearned attachment behaviors seen in insecure individuals (Mikulincer et al. 2003).

Research to date has not examined how attachment representations might predict one's propensity to become flooded during couple conflict. For example, attachment insecurity could relate to how one behaves during conflict (e.g., anxious individual seeking reassurance) resulting in the other partner feeling overwhelmed (e.g., avoidant partner feeling flooded because the approach behavior opposes their goal of achieving distance). Malik et al. (2020) found that flooding predicted both partners' experienced anger during conflict. This supports the interpersonal effects of flooding, such that a partner's attachment insecurity could relate to one's own flooding as well as one's partner's flooding. Moreover, examining these constructs over the transition to parenthood might be particularly relevant, as attachment representations become increasingly salient (Rholes and Paetzold 2019) and conflict often increases in severity and frequency (Kluwer 2010).

### 1.4 Attachment, Flooding, and the Transition to Parenthood

The transition to parenthood is a period of activation and change in multiple domains. The salience of attachment representations resurfaces during parenthood as partners begin caretaking and creating affiliative bonds with their children (Jones et al. 2015). Support for the differential exposure hypothesis (Kessler 1979) suggests that the transition to parenthood might increase relationship conflict in couples through increased stressors (e.g., work‐life balance, childcare, managing finances, etc.; Huss and Pollmann‐Schult 2020). There is also preliminary research to suggest that difficulties managing emotions increase across pregnancy and infancy, but improve during toddlerhood (Grolleman et al. 2023). There is a paucity of research to date that examines interpersonal difficulties in regulating emotions across the transition to parenthood. Researchers have suggested that emotional flooding is a stable, trait‐like characteristic (J. M. Gottman 1993); however, this is largely theoretical. The transition to parenthood might offer a unique window of opportunity to examine attachment representations and how they might be related to fluctuations in emotional flooding as couples navigate this period together.

#### 1.4.1 Gender Differences

The changes that accompany the transition to parenthood are largely gender specific in heterosexual couples. For example, evidence suggests that women experience parenthood as a greater source of conflict than men, which might be due to the increased demands of childcare that often fall on women and declines in partner supportive behavior during this period (Mickelson and Biehle 2017). The increase in conflict intensity and declines in partner supportiveness might indicate a propensity to find conflict overwhelming, especially during the heightened stress that accompanies parenthood.

Research examining gender differences in attachment styles between partners has yielded largely insignificant results (e.g., Velotti et al. 2016). However, there is some evidence that gender differences exist when examining attachment insecurities and emotion regulation difficulties. In a study of premarital heterosexual couples, Velotti et al. (2016) found that women reported greater difficulties in the regulation of negative emotions than men, especially as it related to goal‐oriented behavior (e.g., getting things done while upset). The authors suggest that women who internalize traditional gender norms might judge themselves more harshly for experiencing distress, which might diminish their ability to express their goals to their partner.

Gender differences in emotional flooding have also been theorized. For instance, Gottman and Levenson (1988) speculated that men might become flooded by less intense negative affect and behaviors than women. While J. M. Gottman (1994) found that men were more likely to become flooded and subsequently withdraw during conflict, other researchers have found no gender differences for the propensity to become flooded during couple conflict (Malik et al. 2020). Overall, examining these constructs in relation to gender differences is warranted given the inconsistent findings in the literature to date.

### 1.5 Current Study

The current study aims to extend research during the perinatal period and across parenthood by examining the association between attachment insecurity and a form of interpersonal emotion dysregulation known as emotional flooding. Through a dyadic and interpersonal framework, the study hopes to: (a) further interpersonal attachment theory by examining the combinations of attachment styles between expectant couples and (b) understand how attachment pairings are associated with emotional flooding cross‐sectionally and longitudinally.

### 1.6 Hypotheses

For the purposes of replication, we first tested two of J. M. Gottman's (1994) hypotheses: (a) men will report a higher propensity to flood than women; and (b) the propensity to flood is a relatively stable, trait‐like experience. However, our focus was to extend emotional flooding from an attachment lens, examining how the dyadic theories of attachment apply to emotional flooding. Our specific hypotheses are as follows:

*Men's and women's attachment anxiety and avoidance will be positively associated with their own emotional flooding given that attachment and emotion regulation share similar brain regions (Coan et al*. 2006 *)*.

*Partner effects were expected to relate to emotional flooding (e.g., women's attachment anxiety predicting men's emotional flooding) given the preliminary work on flooding during conflict (Malik et al*. 2020 *), but no specific hypotheses were outlined due to a lack of research in this area*.

In addition to the actor and partner effects, we also sought to examine the interaction effects between different dyadic attachment pairings. Driven by studies confirming the complementary theory of attachment (e.g., anxious/avoidant pairs are associated with greater distress; Ben‐Naim et al. 2013), our specific hypotheses are:

*Anxious/avoidant interactions will be associated with greater flooding. More specifically, we hypothesize that the interaction between avoidant men and anxious women will predict increased emotional flooding in men (J. M. Gottman* 1994 *). The opposite interaction pairing (avoidant women and anxious men) has no specific hypothesis*.

Driven by the similarity theory of attachment (e.g., anxious/anxious similarities and being associated with greater satisfaction; Holmes and Johnson 2009), and recent literature citing the harmful effects in similarities of avoidance (Wang et al. 2022), our specific hypotheses are:

*Similarities in attachment anxiety and dissimilarities in attachment avoidance will be associated with less flooding for both men and women, due to its association with better relationship functioning, suggesting a less cognitively disorganizing conflict environment*.

We also sought to explore how attachment pairings relate to changes in flooding across the transition to parenthood, but no specific hypotheses were outlined.

## 2 Method

### 2.1 Participants

Ninety‐eight couples participated in this study and were first assessed during the third trimester of pregnancy (Time 1). Subsequent assessments occurred 1 year postpartum (Time 2; *N*_women_ = 84, *N*_men_ = 79), 2 years postpartum (Time 3; *N*_women_ = 76, *N*_men_ = 75), and 4 years postpartum (Time 4; *N*_women_ = 39, *N*_men_ = 39). These assessments were recoded to accurately reflect the actual time intervals between assessments. The entire study period spanned from 2008 to 2014. Honorariums were offered at each timepoint ($50 (CAD) at Time 1, $25 (CAD) at Time 2, $50 (CAD) at Time 3, and $50 (CAD) at Time 4). All procedures were approved by the University of Victoria Research Ethics Board (#23‐0472).

At Time 1, the average age of participants was 32.03 years for men (SD = 5.51) and 29.98 years for women (SD = 5.49). Men had an average of 14.77 (SD = 2.38) years of education, whereas women had an average of 15.28 (SD = 2.31) years of education. Couples had been living together for an average of 4.47 years (SD = 3.23). The average income for men was $51,716 (SD = 35,254) and $35,019 (SD = 24,835) for women. In this sample, 69.4% were legally married, whereas 30.6% were unmarried cohabitating. For men, the ethnic composition was predominantly White (89.8%), followed by people of color (e.g., Asian‐Canadian, African‐Canadian, and Latin‐Canadian; 7.1%), and Indigenous (3.1%). Women's ethnic composition was similar, with individuals identifying as White (87.8%), people of color (e.g., Asian‐Canadian, African‐Canadian, and Latin‐Canadian; 10.2%), and Indigenous (2.0%).

### 2.2 Procedure

At Time 1, interested couples contacted the laboratory and were screened by phone. Eligible participants were then scheduled for appointments to attend the research session. The first time of assessment included completing computer questionnaires (separately), participating in an interview, and participating in interactions with one another. The session lasted about 3.5 h.

At Time 2, participants were contacted via email and asked if they wanted to continue participating in the study. Participants were then given a unique link and password to access online questionnaires and were encouraged to complete them separately from their partner. The same follow‐up procedure was used for Time 3 and Time 4.

### 2.3 Measures

#### 2.3.1 Attachment

To assess adult attachment style, participants completed the Experiences in Close Relationships Scale (ECR; Brennan et al. 1998) at Time 1. This questionnaire includes 18 items that measure attachment anxiety (men's *α* = 0.87, women's *α* = 0.89) and 18 items that measure attachment avoidance (men's *α* = 0.90, women's *α* = 0.86). Example items for anxiety include “I'm afraid that I will lose my partner's love,” whereas example items for avoidance include “I find it difficult to allow myself to depend on romantic partners.” All items were rated on a 7‐point Likert scale from 1 = *disagree strongly* to 7 = *agree strongly*. Items measuring anxiety and avoidance are separately averaged to produce two dimensions of attachment. Higher scores on either dimension represent greater attachment insecurity. Lower scores on each dimension represent a more secure attachment style.

#### 2.3.2 Flooding

The Intimate Partner Flooding Scale (IPFS; Foran et al. 2020) is a 15‐item questionnaire that measures flooding, or the feeling of being overwhelmed by a partner's anger. Participants rated their responses from 1 = *never* to 5 = *almost always*. A higher total score indicates a greater propensity to become flooded by one's partner's anger. Example items include “I feel paralyzed during my partner's angry outbursts” and “My partner's anger leaves me feeling disorganized and stressed.” Based on a previous study, only 9 of the 15 items were retained due to construct validity (Foran and Slep 2007). Six items were removed due to higher‐than‐recommended inter‐item correlations, suggesting redundancy rather than a failure to capture the construct (Clark and Watson 1995). The retained items reflect core aspects of flooding, specifically emotional overwhelm and perceived unpredictability during conflict. The reliability in the current study was excellent, with Cronbach's alpha ranging from 0.91–0.93 for men and from 0.91–0.95 for women.

### 2.4 Data Analysis Plan

Actor partner interdependence models (APIM; Cook and Kenny 2005) were employed using Hierarchical Linear Modeling (HLM; Raudenbush et al. 1995) via HLM 7 software (Garson 2013) which allows dyadic relationships between predictor and outcome variables to be examined. This model takes into account the interdependence between partners in a relationship, incorporating two‐person relationships with appropriate statistical techniques to account for this nonindependence. Statistically speaking, APIM allows researchers to examine actor effects (effect of an individual's attachment on their own emotional flooding), partner effects (the effect of an individual's attachment on their partner's emotional flooding), and actor‐partner interactions (the moderation of each person's attachment on one's emotional flooding).

A dyadic model was created by fitting each participant's data with an intercept and slope. These intercepts and slopes were modeled separately for men and women within couples, following procedures to model longitudinal two‐level dyad data (Raudenbush et al. 1995). The first equation demonstrates the general form of this two‐level model:

$$\begin{array}{c} \text{Level}\;1:{\mathrm{FLO}}_{\mathrm{ij}}\;=\pi_{1j}\times \left(\mathrm{men}\right)\\ \;+\pi_{2j}\times \left(\text{women}\right)\\ \;+\pi_{3j}\times \left({\mathrm{men}}^{'}s\;\text{linear time}\right)\\ \;+\pi_{4j}\times \left({\text{women}}^{'}s\;\text{linear time}\right)\\ \;+\varepsilon_{\mathrm{ij}} \end{array}$$

$$\begin{array}{l} \text{Level}\;2:\pi_{1j}=\beta_{10}+r_{1j}+\left[\text{predictors}\right]\\ \pi_{2j}=\beta_{20}+r_{2j}+\left[\text{predictors}\right]\\ \pi_{3j}=\beta_{30}+r_{3j}+\left[\text{predictors}\right]\\ \pi_{4j}=\beta_{40}+r_{4j}+\left[\text{predictors}\right] \end{array}$$

where FLO_*ij*_ is the level of emotional flooding for an individual partner of a specific couple. The initial levels for partners (*π*_1*j*_ and *π*_2*j*_) and changes over time (*π*_3*j*_ and *π*_4*j*_) were modeled separately by gender. Level 2 equations specify between dyad variations in the coefficients in level 1. We chose to include only linear time in our model, based on previous research suggesting that four time points are often the minimum required to assess quadratic change (Biesanz et al. 2004). Additionally, there was a lack of theoretical justification for non‐linear change in our data, and our sample size aligns more closely with the recommendations for assessing quadratic growth with six time points (Diallo et al. 2014). Furthermore, including quadratic terms raised concerns about overfitting due to negative variances, indicating potential instability in the estimation process.

In order to examine the predictive effects of attachment on flooding, we took a multi‐step approach. First, each of the actor and partner attachment styles was added at level 2 on the intercepts to examine the unique contribution of each person's attachment dimension (either anxiety or avoidance) while controlling for one's other attachment dimension and one's partner's attachment on flooding. Next, attachment interactions between partners (e.g., men's avoidance × women's anxiety) were added at level 2 on the intercepts. Finally, attachment interactions between partners were added at level 2 on the slopes to examine the association between attachment insecurity and flooding across the transition to parenthood.

## 3 Results

### 3.1 Preliminary Analyses

Prior to conducting analyses, measures were examined for outliers, missing data, and potential kurtosis. Logistic regression analyses were conducted in R Studio (v.4.2.1) using the glm function (R Core Team 2022) to predict patterns of missingness at each time point. No demographic variables (e.g., age, education level, marital status, income) were significantly associated with dropout rates.

For both men and women, the sampling distributions for attachment anxiety, attachment avoidance, and flooding were positively skewed. All measures were evidenced as being kurtotic in their distribution. However, the decision to forego transformation to correct skewedness and kurtosis in the data was made based on most of the measures being within the acceptable limits (Kline 2023) and to assist with meaningful interpretation of the results.

Zero‐order correlations were computed for all variables (e.g., attachment insecurity at Time 1, flooding at Times 1–4), separated by men and women (see Table 1 and Table 2). Correlations were generally in line with hypotheses.^1^ In terms of demographic variables, only the level of education was significantly associated with attachment anxiety (*r* = −0.21, *p* = 0.036) and flooding (*r* = −0.26, *p* = 0.009) in women. Additionally, these demographic variables were not found to be related to changes in flooding over time. As a result, we decided not to include them in our final models.

**TABLE 1:** Summary of all intercorrelations for men.

Variable	*M*	SD	1	2	3	4	5
1. Anxiety	39.11	15.04					
2. Avoidance	32.48	14.69	0.33** [0.14, 0.49]				
3. Flooding Time 1	17.39	7.21	0.54** [0.38, 0.67]	0.35** [0.17, 0.51]			
4. Flooding Time 2	17.97	7.86	0.42** [0.22, 0.59]	0.24* [0.02, 0.44]	0.63** [0.47, 0.74]		
5. Flooding Time 3	17.56	7.81	0.44** [0.24, 0.61]	0.22 [−0.01, 0.42]	0.49** [0.29, 0.64]	0.73** [0.60, 0.83]	
6. Flooding Time 4	17.82	7.26	0.29 [−0.03, 0.55]	0.44** [0.15, 0.67]	0.58** [0.32, 0.76]	0.61** [0.35, 0.78]	0.70** [0.49, 0.84]

*Note:* *N* = 98 unless otherwise noted. *M* and SD are used to represent mean and standard deviation, respectively. Values in square brackets indicate the 95% confidence interval for each correlation. The confidence interval is a plausible range of population correlations that could have caused the sample correlation (Cumming 2014). Time 2 *N* = 79; Time 3 *N* = 75; Time 4 *N* = 39.

* *p* < 0.05.

** *p* < 0.01.

**TABLE 2:** Summary of all intercorrelations for women.

Variable	*M*	SD	1	2	3	4	5
1. Anxiety	46.35	17.59					
2. Avoidance	26.64	9.94	0.45** [0.27, 0.59]				
3. Flooding Time 1	16.10	7.01	0.68** [0.55, 0.77]	0.40** [0.22, 0.55]			
4. Flooding Time 2	15.17	6.80	0.52** [0.34, 0.66]	0.28** [0.07, 0.47]	0.69** [0.56, 0.79]		
5. Flooding Time 3	15.76	6.78	0.60** [0.43, 0.73]	0.31** [0.09, 0.50]	0.75** [0.64, 0.84]	0.82** [0.73, 0.88]	
6. Flooding Time 4	16.41	8.12	0.54** [0.27, 0.73]	0.18 [−0.14, 0.47]	0.58** [0.32, 0.76]	0.71** [0.50, 0.84]	0.76** [0.58, 0.87]

*Note:* *N* = 98 unless otherwise noted. *M* and SD are used to represent mean and standard deviation, respectively. Values in square brackets indicate the 95% confidence interval for each correlation. The confidence interval is a plausible range of population correlations that could have caused the sample correlation (Cumming 2014). Time 2 *N* = 84; Time 3 *N* = 76; Time 4 *N* = 39.

* *p* < 0.05.

** *p* < 0.01.

### 3.2 Gender Differences of Emotional Flooding Across the Transition to Parenthood

We conducted a hypothesis test to examine potential gender differences in average flooding at the birth of their child (intercept). Men's emotional flooding was significantly higher than women's at the time of birth (*χ* ^2^ = 7.76, *p* = 0.006). This is consistent with the hypothesis that men have a higher propensity to flood compared to women.

Results from the model indicate that there was no significant change in the slope of flooding over time for either men (*B* = 0.023, *p* = 0.283) or women (*B* = −0.005, *p* = 0.796), which was consistent with the hypothesis that flooding is stable over time. However, in the model containing random effects for slope, there were significant random effects for men (*τ*_30_ = 0.014, *p* = 0.001), accounting for between dyad variation in the slopes for men. This was not the case for women (*τ*_40_ = 0.006, *p* = 0.111).

### 3.3 Actor and Partner Effects at Baseline

After adding each individual's and their partner's attachment styles (attachment anxiety and avoidance) to the model of initial flooding levels, we found a gender difference in attachment anxiety, such that anxiety was positively associated with flooding in men (*B* = 0.217, *p* < 0.001), but not in women (*B* = 0.037, *p* = 0.306) at the third trimester. This was confirmed using a Wald test (*χ* ^2^ = 15.07, *p* < 0.001), noting a significant effect of gender. There was also a gender difference in partner anxiety, such that men's anxiety was positively associated with women's flooding (*B* = 0.216, *p* < 0.001), but not vice versa (*B* = 0.039, *p* = 0.321); this was also confirmed with a significant Wald test (*χ* ^2^ = 17.37, *p* < 0.001). Main effects of attachment avoidance were not associated with flooding at the third trimester. Results of the main effects on initial levels of flooding are shown in Table S1.

### 3.4 Interactions of Attachment Styles at Baseline

**FIGURE 1:**
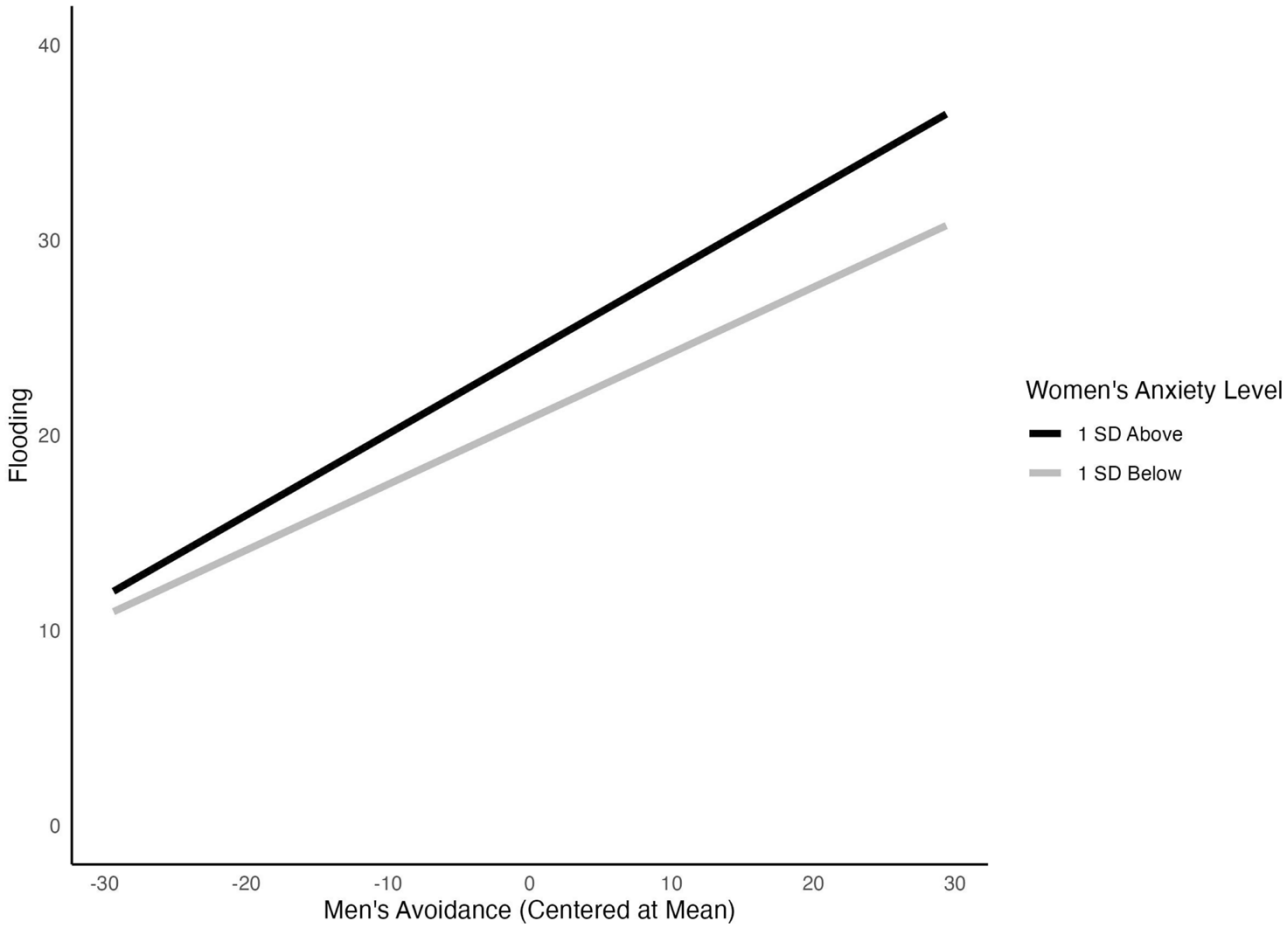
Interaction between men's attachment avoidance and women's attachment anxiety predicting men's emotional flooding at baseline. Men's avoidance is plotted at two standard deviations above and below the mean.

There was a significant interaction term for men between the combination of avoidant men and anxious women (*B* = 0.004, *p* = 0.031) predicting initial levels of emotional flooding during pregnancy. Results are shown in Table S2. We then conducted simple slope tests (e.g., Aiken et al. 1991) to better understand the interaction. This test examines differences in emotion flooding patterns for prototypical partner pairings. Values were plotted so that high corresponded to 1 standard deviation above the grand mean and low corresponded to 1 standard deviation below the grand mean. As shown in Figure 1, although men who were high in avoidance generally reported greater emotional flooding, this association was amplified when their partner was high in anxiety (*B* = 0.241, *p* = 0.001), compared to when their partner was low in anxiety (*B* = 0.098, *p* = 0.03). Similarity interactions between partners (e.g., anxiety/anxiety and avoidance/avoidance pairings) were not significant.

### 3.5 Attachment Pairings and Longitudinal Effects on Flooding

Finally, attachment main effects and interactions were added at level 2 to the slopes of flooding for men and women separately (see Table S3), assessing how attachment insecurity influences longitudinal changes in flooding. Before interpreting the main analyses, a pattern mixture model was employed to evaluate the potential impact of attrition. Specifically, a dummy variable indicating dropout at Time 4 was included as both a main effect and as an interaction term with all Level 2 predictor variables in the longitudinal model. Results indicated that dropout at Time 4 did not significantly predict the slope of flooding for men (*B* = 0.210, *p* = 0.547) or women (*B* = −0.145, *p* = 0.644), nor did it interact significantly with any predictor variables. These findings suggest that the primary results are unlikely to be biased due to differential attrition at Time 4.

**FIGURE 2:**
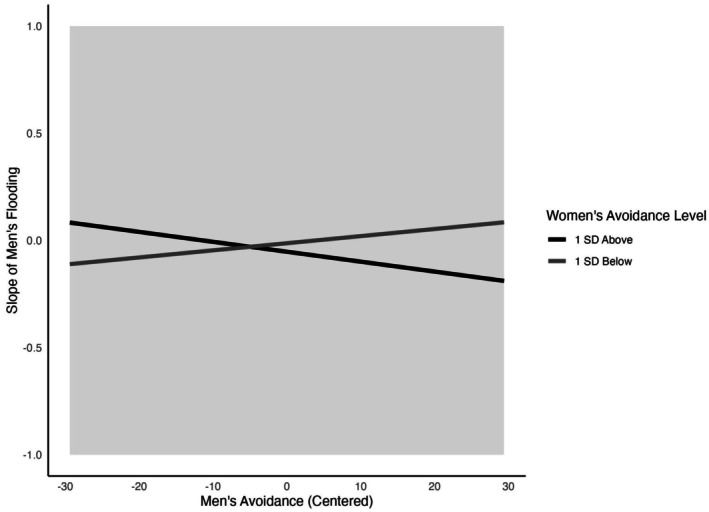
Interaction of men's and women's attachment avoidance on men's change in flooding over time. Men's avoidance is plotted at two standard deviations above and below the mean. Regions of significance are indicated by the gray shaded box.

Men's avoidance and women's avoidance interacted to predict conditional increases in flooding over time in men (*B* = −0.0004, *p* = 0.022). Simple slopes suggested that men's avoidance was associated with increases in flooding over time but was only seen when their partner was low in avoidance (*B* = 0.014, *p* < 0.001), compared to high in avoidance (*B* = 0.003, *p* = 0.419). To better understand the nature of the moderation, we graphically plotted the interaction between men's and women's avoidance and calculated the Proportion of Interaction (PoI; Roisman et al. 2012) and the Regions of Significance (RoS; Dearing and Hamilton 2006). The PoI estimates the proportion of the predictor range (men's avoidance) in which individuals exposed to the susceptibility factor—in this case, men whose partners are low in avoidance—experience better outcomes (i.e., lower increases in flooding over time). The PoI was computed using the squared ratio of simple slopes at ±2 SDs of men's avoidance, yielding a value of 0.33 (Roisman et al. 2012). This indicates that avoidant men experienced smaller increases in flooding over time in approximately one‐third of the predictor range, particularly when their own avoidance was low. The RoS analysis indicated that the interaction was significant across the full range of men's avoidance within two standard deviations. These findings provide partial support for a differential susceptibility effect, as the PoI approaches but does not fully fall within the conventional 0.40–0.60 range consistent with differential susceptibility (Roisman et al. 2012; see Figure 2).

Specifically, low levels of women's avoidance appeared to moderate the association between men's avoidance and changes in emotional flooding over time. When men reported low avoidance, the association with changes in emotional flooding over time appeared weaker, suggesting a more attenuated increase. In contrast, at higher levels of men's avoidance, having a partner low in avoidance was associated with greater increases in emotional flooding over time. No other longitudinal interactions were significant for men or women (e.g., avoidant‐anxious).

## 4 Discussion

The present study advances the literature on attachment and emotion by examining the interpersonal nature of attachment theory and one form of interpersonal emotion dysregulation, emotional flooding, across the transition to parenthood. Our findings stress the importance of examining attachment insecurities between partners and their emotional implications during couple conflict over time, as different combinations of attachment insecurities between partners were associated with flooding prenatally and across parenthood. While we cannot claim that these patterns are exclusive to parenthood, the unique stressors, changes, and challenges associated with this life stage likely provide an important context for understanding how attachment dynamics unfold during this period. These findings underscore the relevance of examining attachment insecurities not just intra‐individually, but in the relational context, where partner dynamics play a crucial role in the emotion (dys)regulation during conflict over time.

Our findings are consistent with previous literature suggesting that men have a greater propensity to flood compared to women. J. M. Gottman (1994) posited that men are flooded by less intense negative affect and behaviors than their partners. However, some recent studies have found no such gender differences, with Foran et al. (2020) for example, finding that men's and women's levels of emotional flooding were nearly identical. Perhaps the differences in findings could be due to the sample composition, as Foran et al. (2020) sample consisted of couples with a child between the ages of 3 and 7 years and may have been in a life stage with less disruption to the attachment system.

We conducted follow‐up analyses in the current study and found that the gender differences in flooding extended to 2 years postpartum, but then were not significant at 4 years postpartum. Expecting a child, and the years that follow, might de‐stabilize couples' relationships, increasing the frequency and severity of conflict. It also might be that the threshold for experiencing conflict as aversive could be lowered due to the increased stress, lack of sleep, and other factors that accompany the arrival of a child. Emotional flooding, especially for expectant fathers, might indicate the use of maladaptive coping strategies to deal with emotions; along with poor regulation strategies, it has been found that expectant fathers also report greater irritability and more negative affect during the prenatal versus postpartum period (Condon et al. 2004). This combination of factors could explain men's heightened flooding prenatally.

During the prenatal period, we found actor and partner effects of attachment anxiety, such that men's anxiety was associated with their own tendency to become emotionally flooded during conflict. In addition, men's anxiety was associated with women's flooding, providing evidence of the interpersonal nature of emotional flooding. Attachment anxiety has been associated with methods that involve hyperactivation of the attachment system (Mikulincer and Shaver 2003), leading to self‐defeating appraisals, pessimistic beliefs about one's ability to manage situations (Shaver and Clark 1994), and rumination of threat‐related events and their elicited emotions (Mikulincer and Shaver 2003). These findings provide support for the association between men's attachment anxiety and both partners' flooding. If an individual feels they are unable to control a situation, especially if they interpret their partner's behaviors as being particularly aversive, this could lead to being overwhelmed and flooded by emotions during conflict. Such cognitive heuristics are common in attachment anxiety, such that anxious individuals' associative memory networks are preconditioned to focus on negative emotional experiences through spreading activation (Main et al. 1985). This can impede one's ability to think about possible remedies, as their attention is focused on the threatening or disruptive nature of the emotional experience at hand. Overall, these biases could perpetuate and amplify emotional flooding during conflict for men.

A partner's attachment insecurity can also impact one's ability to regulate emotions. Attachment anxiety has been negatively associated with one's ability to manage negative emotions during conflict (Creasey et al. 1999) and the tendency to escalate conflict beyond the original issue (Campbell et al. 2005). Therefore, perhaps women in our sample who have partners high in anxiety during the prenatal period become flooded due to the escalation of conflict and their partners' inability to manage emotions during conflict. This escalation could, in turn, lead to feelings of being overwhelmed and finding a partner's behaviors to be unexpected and cognitively disorganizing. In addition, even the mere perception of a partner's attachment anxiety has implications for emotion regulation. Lemay and Dudley (2011) found, in a sample of married or dating couples, that an individual's ability to perceive their partner's attachment anxiety was associated with increased vigilance and affect exaggeration. Using daily diary methods, it was found that being paired with an anxious partner led the target individual to fear upsetting their partner, leading to a concealment of their negative emotions. Other studies have confirmed this association, finding that partners of anxiously attached individuals suppress their emotions instead of pursuing their own needs (Brandão et al. 2020). This suppression of negative feelings could become cognitively taxing over time. In the context of conflict, therefore, an individual's regulatory abilities may be depleted, leading to feeling overwhelmed or cognitively dysregulated by a partner's emotional responses. During the prenatal period, women in our sample may have been particularly susceptible to feeling dysregulated in the context of their partners' anxiety.

In addition, attachment avoidance in men interacted with attachment anxiety in women to predict men's greater emotional flooding during the prenatal period, supporting the complementary theory of attachment. This could be explained by competing emotion regulatory strategies typically employed by differing attachment styles. Specifically, the interaction mirrors demand‐withdraw patterns (Christensen and Heavey 1990), where one partner tends to demand greater intimacy (resembling attachment anxiety), while the other partner withdraws to defend autonomy (resembling attachment avoidance). The present results align with this pattern, although empirical evidence supporting the attachment‐based demand‐withdraw dynamic is limited. For instance, Feeney (1994) found that anxious women paired with avoidant men resulted in lower relationship satisfaction. However, less research has examined how these dynamics relate to perceptions of conflict distress and emotional overwhelm. The subjective interpretation of partner's actions, especially when perceived as overwhelming or unpredictable, may contribute to emotional flooding. Thus, the interactive association between attachment anxiety in women and attachment avoidance in men offers preliminary insights into how demand‐withdraw patterns may manifest in couples, providing further support for the complementary theory of attachment (Holmes and Johnson 2009).

While emotional suppression facilitates the goal of disengagement and detachment, it also limits one's emotional awareness and clarity. This fits with research showing that while avoidant individuals might not be able to directly label their feelings, their internal physiologies indicate otherwise. Mikulincer (1998) demonstrated that avoidant people do not report feeling anger about relational events, but their physiological recordings are indicative of intense anger. Perhaps, then, conflict might not explicitly be mentioned as an aversive experience, but the disconnect between thinking and feeling might lead avoidant individuals to feel overwhelmed in response to their partner's affect; this would be heightened if their partner had an opposing attachment style that is associated with emotional amplification. It has also been found that when involved with avoidant partners, anxiously attached individuals increase emotional suppression, which suggests an override of their preferred emotion regulatory strategies (Winterheld 2016). This could result in compensatory negative affect expression from the anxious individual after trying to suppress, leading to an exacerbated emotional response and increasing the aversiveness of conflict and its overwhelming nature in avoidant individuals. This interaction could also be due to the subconscious perception of one's partner. As mentioned previously, even the mere perception of attachment anxiety in one's partner was associated with maladaptive emotion regulatory processes (Lemay and Dudley 2011).

These results also indicate that interactions between partners with similar attachment orientations (e.g., anxiety/anxiety and avoidance/avoidance) did not predict baseline emotional flooding. This finding is consistent with previous research on relationship functioning, which suggests that direct actor and partner effects of attachment insecurity often account for relationship outcomes. For example, our results, which show that attachment anxiety both predicted men's own flooding (actor effects) and their partner's flooding (partner effects), align with research indicating that main effects of attachment insecurity are related to relationship instability (Conradi et al. 2021). Our study contributes to this literature by examining how these effects impact subjective appraisals of conflict dynamics, which are often linked to lower relationship satisfaction and increased risk of relationship dissolution (Kanter et al. 2022). The lack of support for the similarity hypothesis suggests that partners' similar attachment styles may not necessarily experience additional multiplicative effects on flooding during conflict. In other words, the similarity in attachment orientations might at least prevent the increase in flooding often seen in opposite attachment pairings.

The current study also examined the longitudinal associations of attachment insecurity and flooding across the transition to parenthood. Our findings suggest that emotional flooding may be a relatively stable construct, given that individual attachment insecurity dimensions and most interactions between partners were not associated with changes in flooding over time. Notably, this pattern emerged in a sample of couples transitioning to parenthood, a period of heightened vulnerability marked by significant changes in various relationship domains, including increased life and parenthood‐specific stress (Song‐Choi and Woodin 2021). While the absence of a control group limits direct comparisons, the overall stability of flooding over time may indicate that it functions as a persistent risk factor in couples. This finding aligns with J. M. Gottman's (1994) theory that flooding is a more trait‐like quality. In this context, we found that an avoidant‐avoidant interaction between partners predicted increases in men's flooding, with important nuances depending on the degree of congruence between partners. Although the similarity hypothesis for avoidance has not been consistently supported (Wang et al. 2022), even the incongruence between partners' levels of avoidance did not lead to better functioning. Our results suggest that high levels of avoidance in men, when paired with low avoidance in their partners, contribute to negative outcomes, such as increased flooding over time. Conversely, matching at low levels of avoidance seems to buffer this effect, which is consistent with an attachment security perspective (Mikulincer et al. 2002). Specifically, lower avoidance in both partners, indicating greater attachment security, was associated with less flooding over time.

Attachment avoidance has been associated with a reduced ability to regulate negative affect (Creasey et al. 1999), which could be why men high in avoidance might become flooded based on their partner's reactions during conflict. In addition, past research has demonstrated that individuals high in avoidance make greater negative attributions about their partners' behavior (Gallo and Smith 2001). This could explain avoidant men's propensity to become flooded over time, based on the overwhelming nature of conflict and the misattribution of their partner's intentions leading to emotional intensification. Furthermore, Velotti et al. (2016) found that attachment avoidance was positively associated with men's unwillingness to tolerate negative emotions. Similar to distress tolerance, this gender effect suggests that men in our sample might have a lower threshold for experiencing a partner's negative affect as aversive.

For avoidant men paired with women who are also high in avoidance, the vulnerability to flooding appears reduced. This may be because their strategies align, such as both partners maintaining emotional distance, and because conflict is not perceived as particularly aversive, as its significance is often minimized (J. M. Gottman 1993). Conversely, when avoidant men are paired with women low in avoidance, the contrast between their emotional needs and regulation strategies could heighten their distress. Specifically, women's lower avoidance may lead them to engage in more active emotion regulation, which could conflict with avoidant men's tendencies toward emotional suppression and distancing (Mikulincer and Shaver 2003). This mismatch can leave avoidant men feeling particularly overwhelmed during conflict, and if this pattern persists, it may reinforce an overlearned response like flooding. In contrast, when both partners are low on avoidance, they may be better equipped to use proactive coping mechanisms during conflict, reducing the likelihood of emotional flooding. This is consistent with the idea that more secure individuals typically experience lower distress in response to stress, employ better coping strategies, and maintain more positive expectations about themselves and their partners (Mikulincer et al. 2002).

These findings have implications for the importance of emotion regulation not only at the relationship level, but also at the family level. Paley et al. (2005) demonstrated that fathers' insecure attachment assessed during relationship conflict prenatally predicted less positive and more negative whole‐family interactions postnatally. Screening couples at risk of problematic conflict dynamics could highlight those in need of attachment‐based interventions at the prenatal period, with the effectiveness of interventions potentially extending to the entire family longitudinally.

### 4.1 Limitations and Future Directions

While there were notable strengths in the study, it is not without limitations. Without a control group of non‐prospective parents, we cannot definitively conclude that these findings are specific to the transition to parenthood. Additionally, the absence of measures of potentially confounding variables (e.g., relationship satisfaction, communication patterns) that likely influence this period limits the generalizability of these results. However, the findings still contribute to our understanding of the attachment–emotion dysregulation connection, highlighting how these dynamics unfold against the backdrop of the significant changes and stressors that occur during this transition.

Given that the sample was composed of relatively affluent, heterosexual couples expecting their first child, we are cautious about the generalizability of the findings. The measures were based on self‐report, which introduces the possibility of common method variance and self‐report bias. Participants may have underreported levels of flooding and attachment insecurity due to social desirability or lack of full awareness of their emotional responses. This may be particularly relevant given that the participants endorsed rather moderate levels of attachment anxiety and avoidance. Given that the ECR scale measures attachment along a spectrum, it is worth noting that our participants did not score at the extreme levels of either attachment dimension, indicating more secure attachment styles. The ECR also did not explicitly ask participants to indicate their attachment style in reference to their current relationship partner, which might have further confounded the results. Finally, more sensitive analyses to address similarity effects, such as response surface analysis, might be better situated to examine the matching of anxiety and avoidance in partners. Future studies could employ this technique in relation to couple conflict and emotional flooding to provide a more comprehensive approach to dyadic interaction effects.

In the context of couple conflict, including psychophysiological measures alongside self‐reported emotional flooding could elucidate the potential discrepancy between physiology and cognition. Since there is speculation that this discrepancy is more likely in avoidant individuals (J. M. Gottman 1993), perhaps avoidant individuals are under‐reporting their tendency to become emotionally flooded in this study, which could be corroborated by heart rate variability, skin conductance, and other measures to assess dysregulation. For example, avoidant conflict behavior could be used to influence one's partner negatively (as an attempt to invalidate a partner's feelings by drawing away), downregulate one's own emotional reactions (avoiding as a form of escape from one's own emotions), or both.

## 5 Conclusion

This study provides a glimpse into the complex dynamics of attachment insecurity and its association with flooding across the transition to parenthood. The dyadic nature of the study allowed us to examine the interpersonal emotional mechanisms linking attachment style and flooding within and between partners. Attachment insecurity was associated with increased flooding in various combinations of partners, both cross‐sectionally and longitudinally, with some important gender differences. These findings provide evidence for the complexity of attachment pairings and the need to confirm relevant interpersonal attachment theories in the future (e.g., similarity vs. complementary hypotheses of attachment). These findings suggest that increasing one's ability to effectively regulate emotions during conflict may decrease aversive behaviors in couples, especially in those with attachment vulnerabilities.

## Conflicts of Interest

The authors declare no conflicts of interest.

## Supporting information


**Appendix S1:** famp70057‐sup‐0001‐AppendixS1.docx.

## Data Availability

The data used in the research cannot be publicly shared due to restrictions relating to the privacy of research participants.

## Endnotes

^1^
We also conducted paired sample t‐tests to determine potential gender differences of predictor variables across the four time points. Women reported higher attachment anxiety (t(97) = 3.35, *p* < 0.001) and men reported higher avoidant attachment (t(97) = 3.53, *p* < 0.001) at time 1. Flooding did not differ between men and women at the third trimester (t(97) = −1.29, *p* = 0.11). However, men reported greater flooding 1 year postpartum (t(74) = 2.46, *p* < 0.05), 2 years postpartum (t(69) = 2.39, *p* < 0.05), but not 4 years postpartum (t(36) = 2.00, *p* = 0.24).
