# Supplementary material for: Perceptions of Conflict at the Transition to Parenthood: Exploring Adult Attachment Pairings as Predictors of Emotional Flooding
Source: Fam Process. 2025 Jul 30;64(3):e70057. doi: 10.1111/famp.70057 (PMC12310420; doi:10.1111/famp.70057)
Supplement: Supplementary file 1 — Appendix S1: famp70057‐sup‐0001‐AppendixS1.docx. [file FAMP-64-0-s001.docx]

**Supplemental Materials**

**Table S1**

*Actor and Partner Effects of Attachment Insecurity Predicting Initial Levels of Flooding*

| Predictor | Estimate | | *SE* | *t (df)* | *p* |
| --- | --- | --- | --- | --- | --- |
|  |  |  |  |  |  |
| **Man Flooding** |  |  |  |  |  |
| Intercept | 18.01 |  | 0.55 | 32.97 (91) | <.001 |
| Men’s avoidance | 0.27 |  | 0.12 | 2.31 (91) | .023 |
| Men’s anxiety | 0.17 |  | 0.09 | 1.81 (91) | .074 |
| Women’s avoidance | -0.15 |  | 0.16 | -0.93 (91) | .354 |
| Women’s anxiety | 0.17 |  | 0.07 | 2.35 (91) | .021 |
| Men’s avoidance x women’s anxiety | .004 |  | .002 | 2.19 (91) | .031 |
| Men’s anxiety x women’s avoidance | .001 |  | .003 | .579 (91) | .564 |
| **Woman Flooding** |  |  |  |  |  |
| Intercept | 15.93 |  | 0.48 | 33.00 (91) | <.001 |
| Women’s avoidance | -0.01 |  | 0.07 | -0.16 (91) | .875 |
| Women’s anxiety | 0.04 |  | 0.08 | 0.458 (91) | .648 |
| Men’s avoidance | 0.03 |  | 0.13 | 0.22 (91) | .824 |
| Men’s anxiety | 0.18 |  | 0.06 | 3.11 (91) | .002 |
| Women’s avoidance x men’s anxiety | .001 |  | .001 | .809 (91) | .420 |
| Women’s anxiety x men’s avoidance | .0001 |  | .002 | .038 (91) | .969 |

**Supplemental Table 2**

*Actor, Partner, and Interaction Effects Predicting Initial Levels of Flooding*

| Predictor | Estimate | | *SE* | *t (df)* | *p* |
| --- | --- | --- | --- | --- | --- |
|  |  |  |  |  |  |
| **Man Flooding** |  |  |  |  |  |
| Intercept | 18.01 |  | 0.55 | 32.97 (91) | <.001 |
| Men’s avoidance | 0.27 |  | 0.12 | 2.31 (91) | .023 |
| Men’s anxiety | 0.17 |  | 0.09 | 1.81 (91) | .074 |
| Women’s avoidance | -0.15 |  | 0.16 | -0.93 (91) | .354 |
| Women’s anxiety | 0.17 |  | 0.07 | 2.35 (91) | .021 |
| Men’s avoidance x women’s anxiety | 0.004 |  | .002 | 2.19 (91) | .031 |
| Men’s anxiety x women’s avoidance | .001 |  | .003 | .579 (91) | .564 |
| Men’s anxiety x women’s anxiety | -.002 |  | .003 | -.755 (91) | .452 |
| Men’s avoidance x women’s avoidance | -.004 |  | .004 | -1.09 (91) | .281 |
| **Woman Flooding** |  |  |  |  |  |
| Intercept | 15.93 |  | 0.48 | 33.00 (91) | <.001 |
| Women’s avoidance | -0.01 |  | 0.07 | -0.16 (91) | .875 |
| Women’s anxiety | 0.04 |  | 0.08 | 0.458 (91) | .648 |
| Men’s avoidance | 0.03 |  | 0.13 | 0.22 (91) | .824 |
| Men’s anxiety | 0.18 |  | 0.06 | 3.11 (91) | .002 |
| Women’s avoidance x men’s anxiety | .001 |  | .001 | .809 (91) | .420 |
| Women’s anxiety x men’s avoidance | .0001 |  | .002 | .038 (91) | .969 |
| Women’s anxiety x men’s anxiety | -.0006 |  | .0015 | -.389 (91) | .698 |
| Women’s avoidance x men’s avoidance | .0024 |  | .0026 | .928 (91) | .356 |

**Supplemental Table 3**

*Actor, Partner, and Interaction Effects Predicting Changes in Flooding Over Time*

| Predictor | Estimate | | *SE* | *t (df)* | *p* |
| --- | --- | --- | --- | --- | --- |
|  |  |  |  |  |  |
| **Man Flooding** |  |  |  |  |  |
| Intercept | 17.69 |  | 0.56 | 31.37 (89) | <.001 |
| Men’s avoidance | 0.14 |  | 0.11 | 1.03 (89) | .306 |
| Men’s anxiety | 0.28 |  | 0.14 | 2.01 (89) | .047 |
| Women’s avoidance | -0.14 |  | 0.20 | -0.71 (89) | .481 |
| Women’s anxiety | 0.15 |  | 0.11 | 1.38 (89) | .171 |
| Men’s avoidance x women’s anxiety | -0.002 |  | 0.003 | -0.70 (89) | .483 |
| Men’s anxiety x women’s avoidance | -0.001 |  | 0.003 | -0.175 (89) | .862 |
| Men’s anxiety x women’s anxiety | -0.001 |  | .002 | -.240 (89) | .811 |
| Men’s avoidance x women’s avoidance | 0.003 |  | 0.006 | 0.464 (89) | .644 |
| **Woman Flooding** |  |  |  |  |  |
| Intercept | 15.95 |  | 0.47 | 33.76 (89) | <.001 |
| Women’s avoidance | 0.003 |  | 0.094 | 0.036 (89) | .971 |
| Women’s anxiety | 0.064 |  | 0.102 | 0.625 (89) | .534 |
| Men’s avoidance | 0.107 |  | 0.188 | 0.570 (89) | .0570 |
| Men’s anxiety | 0.197 |  | 0.100 | 1.98 (89) | .051 |
| Women’s avoidance x men’s anxiety | .001 |  | .002 | .388 (89) | .699 |
| Women’s anxiety x men’s avoidance | -0.001 |  | .003 | -0.228 (89) | .821 |
| Women’s anxiety x men’s anxiety | -0.00 |  | 0.002 | -0.006 (89) | .995 |
| Women’s avoidance x men’s avoidance | -0.00 |  | 0.004 | -0.065 (89) | .949 |
| **Man Flooding Slope** |  |  |  |  |  |
| Slope | 0.02 |  | 0.02 | 1.15 (89) | .254 |
| Men’s avoidance | 0.01 |  | 0.003 | 2.63 (89) | .010 |
| Men’s anxiety | -0.003 |  | 0.005 | -0.657 (89) | .513 |
| Women’s avoidance | -0.002 |  | 0.007 | -0.227 (89) | .821 |
| Women’s anxiety | 0.006 |  | 0.003 | 1.84 (89) | .069 |
| Men’s avoidance x women’s anxiety | -0.00 |  | 0.00 | -0.107 (89) | .915 |
| Men’s anxiety x women’s avoidance | 0.00 |  | 0.00 | 2.33 (89) | .022 |
| Men’s anxiety x women’s anxiety | -0.00 |  | 0.00 | -1.67 (89) | .098 |
| Men’s avoidance x women’s avoidance | -0.0004 |  | 0.0002 | -2.38 (89) | .019 |
| **Woman Flooding Slope** |  |  |  |  |  |
| Slope | -0.007 |  | 0.018 | -0.38 (89) | .706 |
| Women’s avoidance | -0.00 |  | 0.00 | -0.31 (89) | .760 |
| Women’s anxiety | -0.00 |  | 0.00 | -0.14 (89) | .888 |
| Men’s avoidance | -0.01 |  | 0.01 | -1.14 (89) | .258 |
| Men’s anxiety | 0.002 |  | 0.003 | 0.534 (89) | .595 |
| Women’s avoidance x men’s anxiety | -0.00 |  | 0.00 | -0.07 (89) | .948 |
| Women’s anxiety x men’s avoidance | 0.00 |  | 0.00 | 0.75 (89) | .458 |
| Women’s anxiety x men’s anxiety | -0.00 |  | 0.00 | -0.83 (89) | .411 |
| Women’s avoidance x men’s avoidance | 0.00 |  | 0.00 | 0.22 (89) | .826 |
